# Impedance Spectroscopy Analysis of PbSe Nanostructures Deposited by Aerosol Assisted Chemical Vapor Deposition Approach

**DOI:** 10.3390/nano11112817

**Published:** 2021-10-23

**Authors:** Sadia Iram, Azhar Mahmood, Muhammad Fahad Ehsan, Asad Mumtaz, Manzar Sohail, Effat Sitara, Shehla Mushtaq, Mohammad Azad Malik, Syeda Arooj Fatima, Rubina Shaheen, Nasir Mahmood Ahmad, Sajid Nawaz Malik

**Affiliations:** 1 School of Natural Sciences, National University of Sciences and Technology, Islamabad 44000, Pakistan; sadia.iram@sns.nust.edu.pk (S.I.); m.fahad.ehsan@sns.nust.edu.pk (M.F.E.); asad.mumtaz@sns.nust.edu.pk (A.M.); manzar.sohail@sns.nust.edu.pk (M.S.); effat.sitara@sns.nust.edu.pk (E.S.); Shehla.mushtaq@sns.nust.edu.pk (S.M.); 2 Department of Materials, University of Manchester, Manchester M13 9PL, UK; azad.malik@manchester.ac.uk; 3 Central Diagnostic Laboratory, Physics Division, PINSTECH, P.O. Nilore., Islamabad 45500, Pakistan; syedaarooj80@yahoo.com (S.A.F.); rubina_shahin_2003@yahoo.com (R.S.); 4 Department of Materials Engineering, School of Chemical and Materials Engineering (SCME)-National University of Sciences and Technology (NUST), Islamabad 44000, Pakistan; Nasir.ahmad@scme.nust.edu.pk (N.M.A.); sajidnawaz@scme.nust.edu.pk (S.N.M.)

**Keywords:** lead chalcogenide, AACVD, nanostructured deposition, impedance analysis

## Abstract

This research endeavor aimed to synthesize the lead (II) diphenyldiselenophosphinate complex and its use to obtain lead selenide nanostructured depositions and further the impedance spectroscopic analysis of these obtained PbSe nanostructures, to determine their roles in the electronics industry. The aerosol-assisted chemical vapor deposition technique was used to provide lead selenide deposition by decomposition of the complex at different temperatures using the glass substrates. The obtained films were revealed to be a pure cubic phase PbSe, as confirmed by X-ray diffraction analysis. SEM and TEM micrographs demonstrated three-dimensionally grown interlocked or aggregated nanocubes of the obtained PbSe. Characteristic dielectric measurements and the impedance spectroscopy analysis at room temperature were executed to evaluate PbSe properties over the frequency range of 100 Hz–5 MHz. The dielectric constant and dielectric loss gave similar trends, along with altering frequency, which was well explained by the Koops theory and Maxwell–Wagner theory. The effective short-range translational carrier hopping gave rise to an overdue remarkable increase in ac conductivity (σ_ac_) on the frequency increase. Fitting of a complex impedance plot was carried out with an equivalent circuit model (R_g_ C_g_) (R_gb_ Q_gb_ C_gb_), which proved that grains, as well as grain boundaries, are responsible for the relaxation processes. The asymmetric depressed semicircle with the center lower to the impedance real axis provided a clear explanation of non-Debye dielectric behavior.

## 1. Introduction

Nowadays, the microelectronics industry is greatly dependent on miniature electronic components. This has encouraged researchers to explore the electrical conductivity of materials. One recent technological development involved the sensitization of devices with alloys of groups IV–VI element compounds, with photodetection [1,2] and injection laser proficiencies [3], long-wavelength imaging [4,5], diode lasers [6,7], and thermo-photovoltaic energy converters [8]. They also have great technological use in remote sensing [9], thermography [10], chemical gas sensing [11,12], gas spectroscopy, and pollution control [13,14]. Metal chalcogenide nanostructures have a range of extended applications to fabricate ideal infrared radiation detector devices, infrared radiation emitter devices, and solar control coatings [15,16]. Lead chalcogenides (PbSe, PbTe, PbS) are potent candidates for solid-state basic research due to their high-tech standings in the IR field and their exceptional features [17]. Many research groups have focused on PbSe nanostructures because of their semiconducting behaviors; they are also easily available and economically cheap. At room temperature, this compound adopted the cubic phase, having a band gap of 0.27 eV. 

Several techniques are used for synthesis of PbSe, including the photochemical approach [18], sonochemical method [19], atomic layer epitaxy technique [20], pulsed laser deposition method [21], CBD approach [22], vacuum deposition method [23], electrodeposition technique [24], pulse sonochemical approach [25], SILAR [26], as well as aerosol-assisted chemical vapor deposition (AACVD) [27]. Every method has its advantages and disadvantages, structure, and surface morphology; all optoelectronic features rely heavily on the preparation approaches. Therefore, many research groups are exploring the electrical parameters of lead selenide prepared by diverse synthetic approaches. K. I. Udofia and Ikhioya I. Lucky [28] used the electrodeposition method to fabricate PbSe thin films onto ITO substrates and studied the effects of different deposition voltages used (1–5 V); they determined the electrical properties by characterizing the deposited films using the four-point probe technique. The thin films were characterized by a variation in sheet resistivity from 1.50 × 10^4^ (Ωm) to 5.23 × 10^4^ (Ωm) and showed conductivity variation from 1.91 × 10^−5^ (Ωm)^−1^ to 7.40 × 10^−5^(Ωm)^−1^. S. Prabahar and coworkers [29] utilized the vacuum deposition technique to prepare thin films of PbSe on the glass surface; they noticed the variation of electrical properties at the sample thicknesses of 1000 and 2000 Å and at various frequencies (20 Hz–100 KHz) and temperatures (303–483 K). They noticed the variations of the dielectric constant and loss as a function of frequency at various temperatures. They evaluated the temperature coefficients of capacitance and permittivity. Hao Yang [30] and his team synthesized polycrystalline PbSe material by iodine concentration manipulation and noticed that the activation energy of the crystallite boundary barrier could be controlled by varying the iodine concentration. They observed a change that occurred in conductivity, by changing both the temperature (150 to 360 K) and iodine concentration, and discovered that, at 108.2 meV activation energy, a high crystalline boundary barrier was attained. Oluyamo, S. S [31] used a chemical bath deposition to study the pH effect on structural, optical, and electrical parameters of PbSe thin films. They used samples of different thicknesses and noticed that the electrical conductivity, as well as the band gap energy of all observed samples decreased with increasing thickness, whereas the dielectric constant increased with increasing thickness. This shows that, by varying the technological factors in the deposition technique, we can adjust solid-state properties of the materials, and, hence, we can achieve a desired function of the material as well. Feng and coworkers determined electrical and other important properties of PbSe thin films by carrying out sputtering at various pressures [32]; they found that sputtering pressure plays an important role in properties of the PbSe thin films. They also found a decrease in resistivity from 1.6 Ω cm to 0.02 Ω cm, as per the Hall effect measurements, and noticed a decrease in the carrier concentration from 1.19 × 10^20^ cm^−3^ (0.5 Pa) to 4.0 × 10^17^ cm^−3^ (4.0 Pa).

Among the different techniques, the aerosol-assisted chemical vapor deposition (AACVD) is a good way to prepare nanocrystalline metal chalcogenides because they are cheaper and give good yield (without needing expensive instrumentation). Single molecular precursors are well-known for obtaining materials with semi-conduction behavior, by employing the AACVD route [33]. Other advantages of AACVD include the control of the rate of deposition (by regulating the bulk transfer of precursors); flexible reaction conditions, ranging from low pressure to atmospheric pressure, and the products that involve multi-components could also be deposited easily, without disturbing the product stoichiometry [34]. However, AACVD also has a disadvantage—solubility of the precursor in a solvent; if the precursor does not give solubility in a suitable solvent, then it is impossible to utilize AACVD to deposit thin films of that precursor [35]. Deposition of lead chalcogenides with different nanostructures was reported using various single-source precursors, including dichalcocarbamates [36,37], dichalcogenoimidophosphinate lead (II) complexes [27], xanthates [38,39], dichalcogenophosphinates [40,41], dichalcogenophosphates [27], N-acyl selenoureas [42], and metalorganodithiophosphinates [43,44].

In our present contribution, we prepared a single molecular precursor, a lead (II) diphenyldiselenophosphinate complex for the deposition of PbSe nanostructures by the AACVD technique. Mostly, lead selenium metal-organic frameworks are believed to be harmful. However, because of limited research carried out on the lead (II) diphenyldiselenophosphinate complex, any specific literature about its toxicity concerns has not been discovered. AACVD evades this problem and avoids the release of unsafe lead alkyls in comparison to other commonly used techniques, offering continuous deposition of synthesized material vapors via low pressures [43]. Although lead belongs to the family of heavy metals, it is less toxic in lead chalcogenide form as compared to its metallic form. Lead selenide forms a strong ionic bond between Pb and Se atoms to form a cubic crystal lattice. There is a negligible chance of any release of lead or selenium from this crystal lattice where, as in the metallic form, the bonds between Pb and Pb atoms are metallic bonds, which are significantly weaker than ionic bonds.

This work reports employment of lead (II) diphenyldiselenophosphinate for PbSe nanostructures deposited, employing the aerosol-assisted chemical vapor technique along with exploration of its electrical properties. The synthesis of PbSe from the lead (II) diphenyldiselenophosphinate complex is novel, as this complex has been used for the first time as a single source precursor to deposit PbSe thin films by AACVD and, hence, in this investigation of its electrical properties, it was done for the very first time. The only requirement for the AACVD method is that the precursors should be soluble in a solvent system so that a mist is formed by ultrasound, which is then carried by a transporting gas (argon) to the deposition chamber. In this case, lead (II) diphenyldiselenophosphinate was readily soluble in tetrahydrofuran (THF). No residue was observed at the bottom of the flask at the end of experiment, indicating the high solubility of the precursor. In case of partial solubility, the deposited films are very thin due to less transportation of the precursor to the deposition chamber, and there is always a residue found at the bottom of the flask at the end of the experiment. Therefore, purity and solubility are two top requirements for the AACVD method. In general, single source precursors are more pure than the multi-source precursors for most being crystal-line solids. We used lead (II) diphenyldiselenophosphinate, a crystalline solid as a novel single source precursor for the deposition of PbSe, for the first time, by the AACVD method.

## 2. Materials Used

All materials used were bought in purified form from Sigma Aldrich UK and used without carrying out any other purifying step. Chemicals that were used included lead chloride, sodium diphenyldiselenophosphinate, methylbenzene, oxolane, acetone, and ethanol.

### 2.1. Preparation of Lead (II) Diphenyldiselenophosphinate

Preparation of the lead (II) diphenyldiselenophosphinate complex was carried out by the technique conducted by Kutchen W et al. [45]. A dilute solution of sodium diphenyldiselenophosphinate was added slowly to the aqueous solution of lead chloride under continuous stirring, which resulted in the formation of precipitates, which were then filtered and air-dried. Further, crystals of lead (II) diphenyldiselenophosphinate were achieved via a recrystallization approach using methylbenzene and acetone.

### 2.2. AACVD System

AACVD kit charged with a lead (II) diphenyldiselenophosphinate complex was rigged to accomplish the synthesis of nanostructures. In a 100 mL round-bottom flask, containing 10 mL oxolane, we dissolved the 0.2 g (0.8 mmol) of the lead (II) diphenyldiselenophosphinate complex. A carrier gas (argon) was attached with a reactor tube containing six glass slides as substrates. The purpose of using argon gas was to prevent oxidation and to help in the transmission of aerosol (formed within the solution flask) towards the tube reactor. The tube reactor was placed inside a Carbolite laboratory heating chamber furnace. Then, that round-bottomed flask comprising a precursor solution was positioned in the piezoelectric modulator water bath of the PIFCO-Ultrasonic Humidifier machine, model no 1077. The precursor solution was transformed to aerosols, which were swiped to the reactor’s hot-walled zone, where glass slides acted as a substrate, and PbSe depositions were formed on its surface by the decomposition of the complex. The deposition process was made at 400 and 450 °C for 1 h along with a 200 SCCM flow of argon gas at atmospheric pressure. The obtained depositions were a greyish black powder of PbSe attached to the glass surface.

### 2.3. Dielectric and Impedance Studies

Good impedance features were believed to be given by the depositions, with an equal ratio of all involved reactants. Thus, PbSe fabricated by a 450 °C deposition procedure was used to perform impedance analysis as its EDX confirmed an almost equal Pb and Se ratio.

Pellets were formed by scratching the PbSe powder from the glass surface and then pressing it into a pellet, with a diameter of 13 mm and thickness of 1.3 mm. Afterward, pellets were sintered in the heating furnace, at a 160 °C temperature for 4 h. Dielectric and impedance analysis was attained over the frequency range of 100 Hz–5 MHz at room temperature, with the help of a Precision Impedance-Analyzer (Wayne Kerr 6500B series).

### 2.4. Instrumentation

Mass spectral analysis was carried out via a micro mass Micromass Autospec Q OPUS software instrument. An electron beam of impact energy at 70 eV at 10 y7 Torr was employed to start the fragment formation. TGA and the elemental analysis were obtained from the microanalysis laboratory at the University of Manchester, with an elemental analyzer Thermo Scientific Flash 2000 and Seiko SSC/S200 in an N_2_ environment. TGA analysis was performed, from room temperature to 600 °C, setting the heating rate at 10 °C per min. A Bruker D8-Advance diffractometer fitted out by a Cu-Kα source was employed to attain XRD results. Morphological analysis was acquired via an FEI.XL-30-SEM instrument. Transmission electron microscopy was collected using Tecnai, F30-FEG TEM equipment. Impedance studies were carried out with the help of a Precision Impedance Analyzer (Wayne Kerr 6500B series). Fitting of the observed results were conducted via ZView software.

## 3. Results and Discussion

### 3.1. Analysis of Lead (II) Diphenyldiselenophosphinate Complex

Elemental analysis report (Figure S1) for C_24_H_20_P_2_PbSe_4_ (M.W = 893.41) found that C (32.27%), Pb (23.19%), H (2.26%), P (6.93%), and Se (35%) while experimental values were C (32.48%), H (2.22%), Pb (22.90%), P (6.93%), and Se (35%). FTIR (Figure S2): 3049 cm^−1^ ʋ(Ar-CH), 747.80 cm^−1^ ʋ(Pb-Se), and 685.28 cm^−1^ ʋ(P-C). Mass spectroscopy (Figure S3): M+ *m*/*z*= 894.7531 [PbP_2_Se_4_(C_6_H_5_)_4_], *m*/*z* =261 [SeP(C_6_H_5_)_2_], and *m*/*z* =342.2 [Se2P(C_6_H_5_)_2_].

### 3.2. Structural and Gravimetric Profile

**Figure 1:**
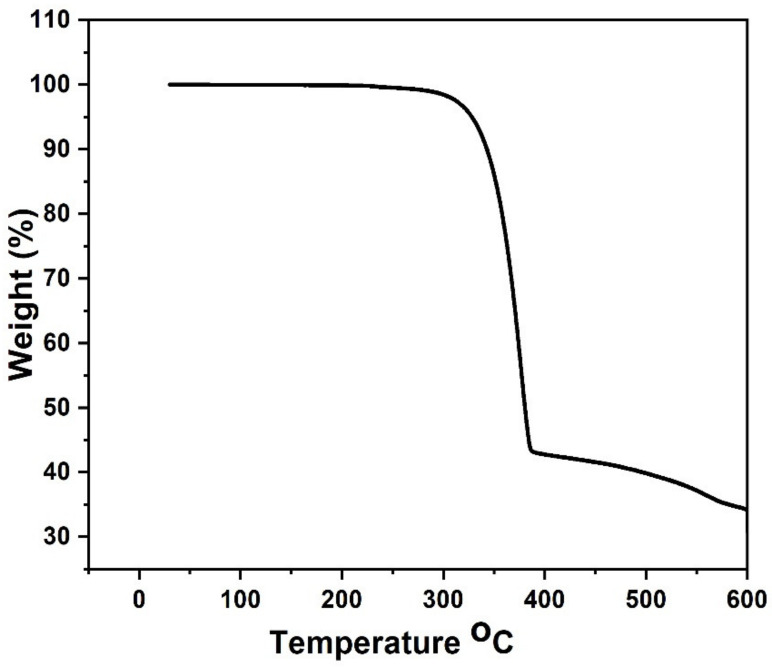
Thermogravimetric runs of the lead (II) diphenyldiselenophosphinate complex.

TGA profile of [Pb(iPh_2_PSe_2_)_2_] showed two-step decompositions between 220 and 380 °C, at 10 °C per min under an N_2_ environment (Figure 1). The major decomposition occurred in the first step, which started at 320 °C and completed at 380 °C. The second decomposition step was slow, starting from 380 °C and carrying on to 600 °C, leaving a residue of about 35%. The calculated value for PbSe was 32%, which was slightly lower than the observed value of the residue. As the graph shows, the baseline is not flat, indicating continuous decomposition after 600 °C.

**Figure 2:**
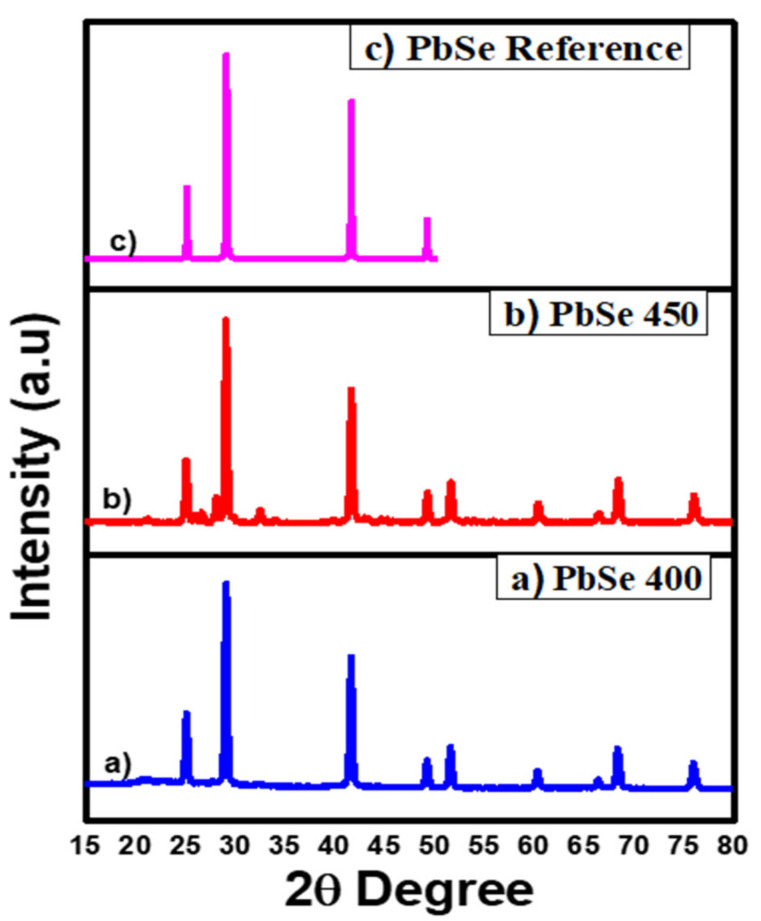
XRD pattern of PbSe prepared at (**a**) 400 °C; (**b**) 450 °C; (**c**) PBSe reference, reproduced or adapted from [47], with permission from Elsevier, 2021.

**Figure 3:**
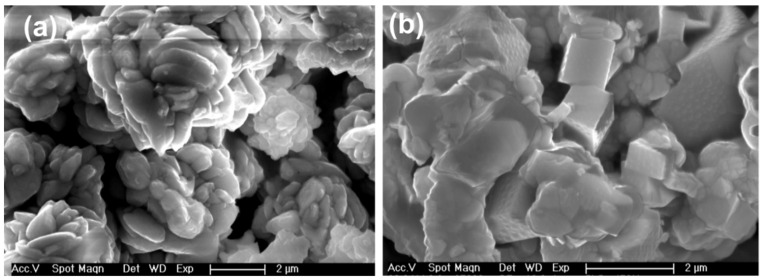
SEM micrographs of PbSe prepared at: (**a**) 400 °C (**b**) 450 °C.

The X-ray diffraction (XRD) spectra of prepared PbSe were acquired using Cu-Kα, an X-ray source having a voltage of about 40 kV with a current supply of 30 mA. The pure cubic phase was indexed by diffraction peaks for both PbSe samples [46] obtained at 400 and 450 °C, JCPD no. 03-065-0693 and 03-065-2941. The absence of additional peaks in the diffraction pattern confirmed the purity of obtained PbSe, as shown in Figure 2. The surface analysis of PbSe thin films by SEM imaging clearly shows three-dimensionally grown interlocked or aggregated nanocubes of PbSe, which were compact and uneven throughout the deposited area, with approximate sizes within 100–200 nm. The presence of faceted crystal shapes is a clear indicator of high crystallinity of synthesized PbSe products. Previously, this general relation between faceted crystal shapes and their crystallinity was observed in different materials [46] Films deposited at a temperature of 450 °C displayed a network of cubes assembled in the form of randomly shaped clusters. The overall morphology of films deposited at 400 °C comprises of a network of clusters formed from the small plate-like crystallites assembled in the shape of different sizes of flowers (Figure 3). This apparent difference in morphology is credited to a difference in deposition temperature, which is considered a significant factor that affects morphology, and is confirmed in our case. EDX analysis (Figure S4) ensured the presence of lead and selenium in ratios 55.58:44.42 (400 °C) and 52.35:47.65 (450 °C), respectively. The EDX results show that the films are selenium deficient.

**Figure 4:**
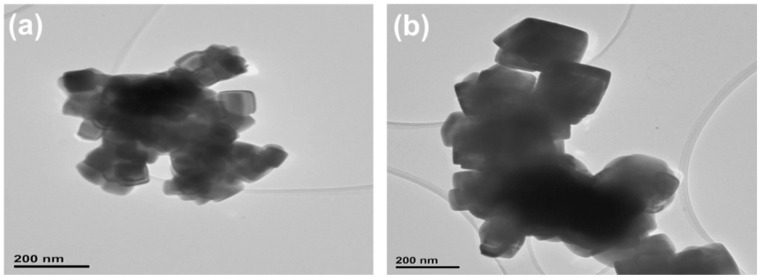
TEM analysis of PbSe samples prepared at (**a**) 400 °C; (**b**) 450 °C.

TEM micrographs of the acquired PbSe at two studied temperatures are represented in Figure 4a,b. The TEM analysis reveals that the films are composed of lead selenide nanocubes of varying sizes, with an average size lesser than 200 nm. Certain nanoparticles are isolated; however, many are in clusters, by nanoparticles; the nanocube morphology agrees with the SEM analysis findings.

### 3.3. Dielectric Constant Behavior

Dielectric constant related to ceramic materials are, reasonably, a complex property, usually denoted via the equation:

ε = ε′ − jε″(1)

**Figure 5:**
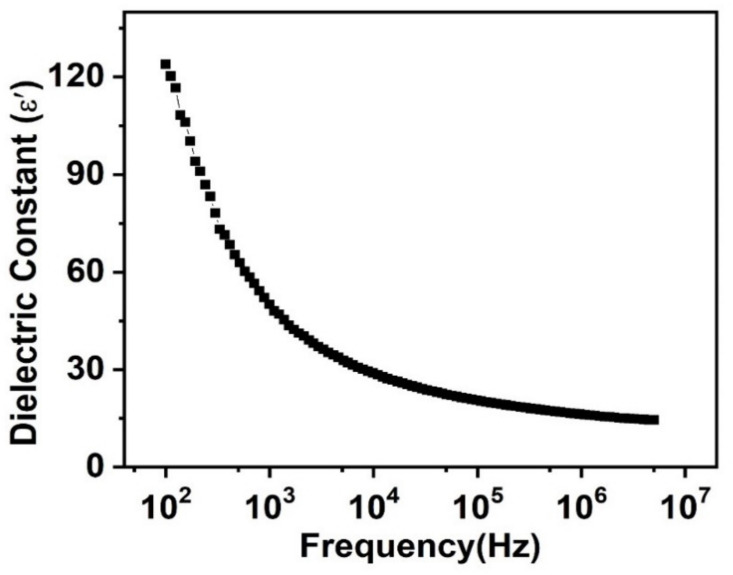
Change of dielectric constant of prepared PbSe along with altering frequency.

Here, ε″ is the imaginary part related to energy dissipation and ε′ is the real part representing energy storage as well as polarization. It is dependent upon interfacial and dipolar polarization ability at low frequency, and other parameters, such as preparatory techniques [48], structural variation [49], chemicals homogeneity [50], variation in dopant substitution [51], distribution of cations [52], alternation of sintering temperature [53], the role of powder density [54], and the effect of porosity [55]. Figure 5 represents the change in the dielectric constant of PbSe along with alternation in frequency. All measurements were executed at room temperature over the frequency range of 100 Hz–5 MHz. Figure 5 shows that, while alternating frequency, ε′ of PbSe shows a maximum value in the lower frequency part; however, it shows a decline with the frequency increase, until it becomes constant after a specific high frequency is attained. The Koops phenomenological model as well as the Maxwell–Wagner polarization provides a reason for this higher dielectric constant observed at the lower frequency region. Dielectric materials usually contain poorly conducting grain boundaries as well as good conducting grains. The reduction in the dielectric constant and the elevating frequency is also the outcome of the decreased effect of the space charge polarization and the delay of the time used to create a polarization, using hopping electrons created among charge carriers. With the increase of frequency, the hopping electron’s frequency simply holds up behind the externally applied electric field, because synchronization, as rapid as alternating field frequency, is not possible for them. This resulted in the decrease of overall polarization, which explains why the dielectric constant showed a reduction in the higher frequency range. This polarization could also be explained by the Maxwell–Wagner effect. Conversely, in multiphase systems, the charge carriers move in different phases and, during this movement, the charge carriers are trapped in interfaces; consequently, the distortion in the electric field occurs along with the rising dielectric constant [52]. The observed dielectric constant is higher than the value reported for the PbSe thin film of 2000 Å thickness at room temperature [29].

### 3.4. Dielectric Loss Behavior

**Figure 6:**
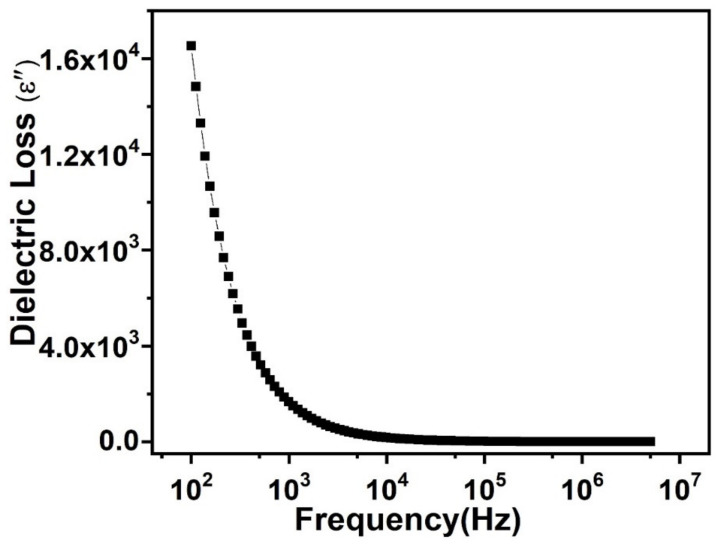
Dielectric loss of prepared PbSe as a function of frequency.

The dielectric constant imaginary part is dielectric loss (ε″) and it is considered an extent of loss of energy taking place in a dielectric medium. Figure 6 shows the effect of the alternating frequency on ε″.

The result shows a similarity with the result by the dielectric constant, an increasing trend in the lower frequencies and nearly “flat: once the definite limiting value of the elevated frequency is attained. This increasing dielectric loss at a low frequency range is correlated to the charge lattice defect shown by space charge polarization at grain boundaries. This lower frequency region corresponds to grain boundaries that are highly resistive and, thus, more energy is required by electrons for hopping among charge traps. Additionally, the existence of crystal defects, as well as imperfections, are the reason for the delayed response shown by polarization dipoles for their orientation, along with fluctuating ac field direction; thus, showing additional energy loss at lower frequencies.

### 3.5. Behavior of Tangent Loss

**Figure 7:**
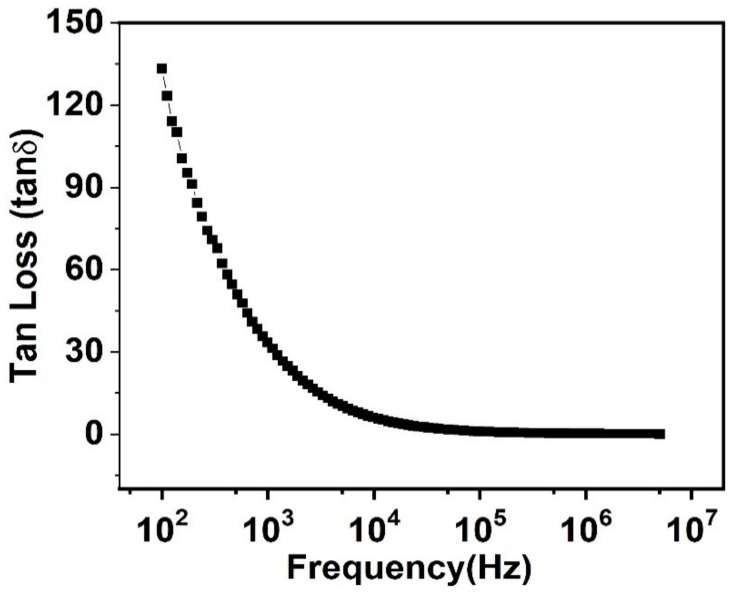
Change of tangent loss of prepared PbSe along with the change of frequency.

The alteration of tan δ alongside the frequency is shown in Figure 7. This shows a high value of tan δ at a lesser frequency, which decreases exponentially with increasing frequency. These smaller values of tan δ, as well as the dielectric constant at elevated frequencies, show impacts in photonic and electro-optic devices [56]. This is conferred already above that, at an elevated frequency—grains along with grain boundaries retain more resistance. Thus, the hopping electron requires more electrical energy in lower frequency parts, whereas at elevated frequencies, the resistance shown by grains is smaller, because of reduced hopping, resulting in less energy intake [57].

### 3.6. The ac Conductivity (σ_ac_)

The ac conductivity can be calculated using the following relation:

(2) $$\sigma =\left[\frac{Z^{\prime }}{Z^{\prime^{2}}+Z^{{\prime \prime }^{2}}}\right]\times \left(\frac{t}{A}\right)$$

where *t* and *A* are the thickness and area of the sample, respectively.

**Figure 8:**
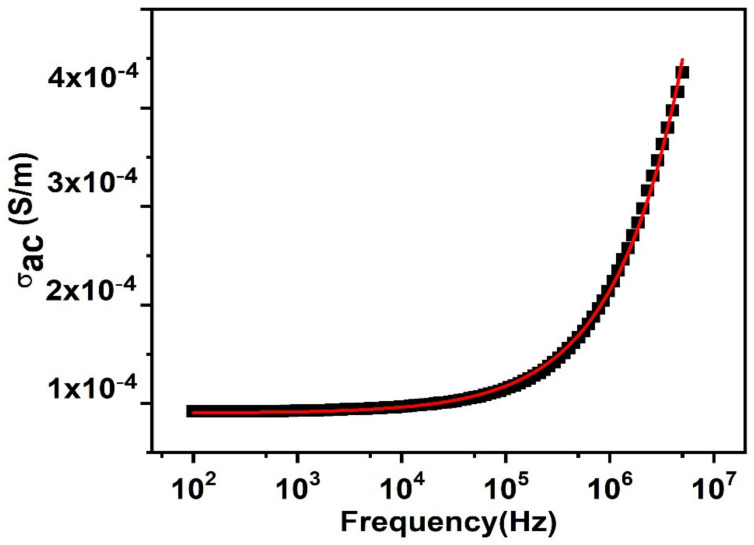
Change in ac conductivity of prepared PbSe along with the change of frequency.

The room temperature variation in ac conductivity behavior, along with the change in frequency is presented in Figure 8. We could observe two types of frequency regions in the figure—lower and higher frequency ranges. The lower frequency range is the frequency independent zone because it describes the successful ion hopping, because of the accessibility of the extended period at a low frequency. Figure 8 clearly shows that, at lower frequencies, the conductance is independent of frequency; its behavior is similar to dc conduction, but at a certain frequency portion (characteristic relaxation frequency). The ac conductivity started a non-linear elevation along with frequency (dispersive region). The jump relaxation model explains this type of behavior, shown by conductivity, which reveals that effective hopping of ions to the nearby vacant sites results in long-range translational motion at lower frequencies. The higher frequency range passes through two kinds of mechanisms, effective hopping, where the ions reach a new site after hopping, and ineffective hopping, where the ions reach back to the state where they started hopping. Throughout this frequency range, these two relaxation processes compete and the rise or decline in the ratio of effective or ineffective hopping finalizes this dispersive part, i.e., the range identifies the two opposing phenomena—short range translational and reorientation phenomena. To assess the dc electrical conductivity, as well as power-law exponent “s”, the Jonscher power law was used to fit the frequency dependent ac conductivity:

(3) $$\sigma_{\left(\omega \right)}=\sigma_{dc\;}+A\omega^{s}$$

where σ_*dc*_ and *A*ω^s^ signify the dc electrical conductivity and frequency-dependent ac conductivity. The value of s ≤ 1 suggests short-range translational hopping motion of charge carriers and s ≥ 1 corresponds to the localized or reorientation hopping mechanism [58]. The fitted parameters are displayed in Table 1. For the present material, the value of frequency exponent “s” deduced from fitting suggests the occurrence of short-range translational hopping. The value of dc conductivity estimated from Jonscher’s fitting is of the same order of magnitude as observed for PbSe thin films deposited onto the ITO substrate [59]. 

### 3.7. Complex Electric Modulus

The electric modulus formalism describes the electrical properties of the materials as it can separate the components showing the same value of resistance and dissimilar values of capacitance. The real and imaginary parts of the electric modulus could be premeditated, using impedance data by the following equations:

(4) $$M^{\prime }=\omega \;C_{0}\;Z^{\prime \prime }$$

(5) $$M^{\prime \prime }=\omega \;C_{0}\;Z^{\prime }$$

where ω, being the angular frequency, $C_{0}={є}_{0\;}A/t$, the geometrical capacitance, and є_0_ is the permittivity of free space.

**Figure 9:**
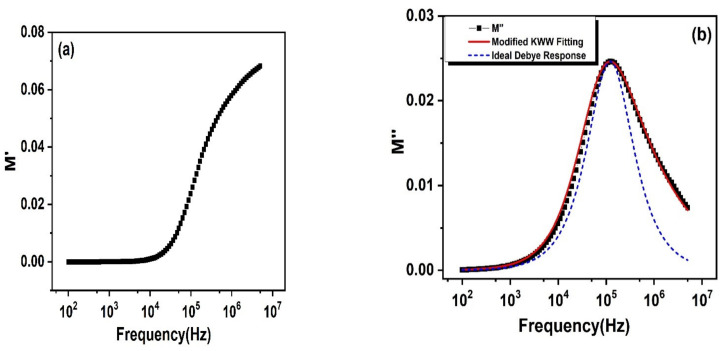

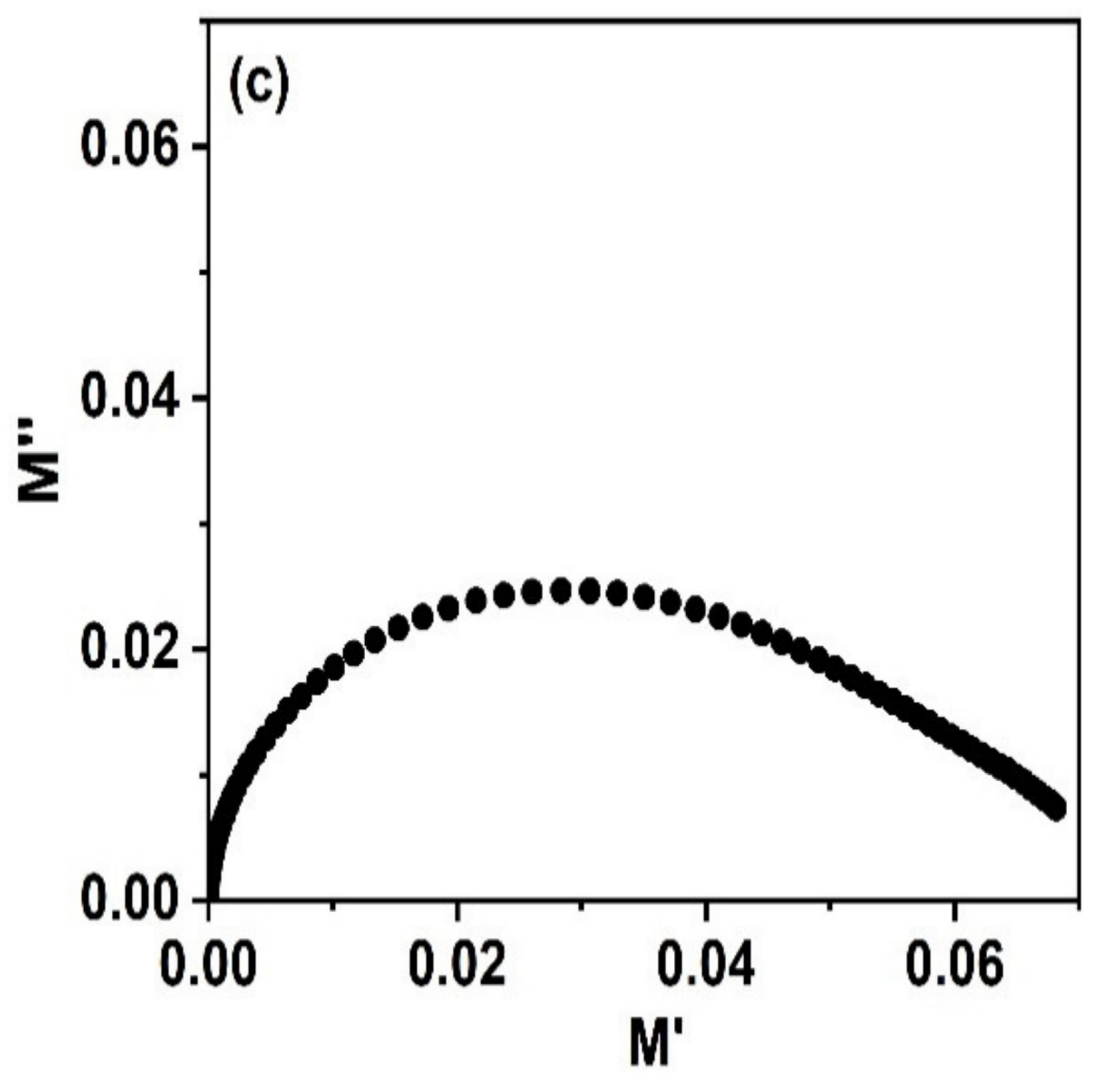
Frequency relying upon the response of (**a**) *M*′; (**b**) *M*″; (**c**) *M*″ verses *M*′ by electric modulus to study the reliance of electrical response on grains plus grain boundary of PbSe.

The frequency relying change of *M*′ is the real part related to the electric modulus observed at room temperature and it is displayed in Figure 9a. It is clear from the figure that the lesser frequency part shows a zero value of *M*′ for the prepared PbSe. This supports the existence of an insignificant electrode polarization effect. With elevating frequency, a sigmoid rise in *M*′ is noticed, as shown in Figure 9a. The continuous dispersion in *M*′ verses frequency supports the short range mobility of charge carriers.

The frequency relying change of the imaginary part (*M*″) of the electric modulus is shown in Figure 9b. This provides detailed knowledge about the charge transport phenomenon, such as conductivity dynamics in charge relaxation, and altered the frequency, electric charge transport, and their differences. This relies on many parameters, such as disorders, defects, microstructures (grain/grain boundary), dielectric spins, and cationic distribution, etc.

The *M*″ plot of as-synthesized PbSe displays a well-defined peak arising on a characteristic frequency region, noteworthy asymmetry seen in the obtained *M*″ trend, a little peak widening, then a peak shifting in the direction of the lesser frequency. The origination of asymmetric behavior is the result given by the conduction/relaxation phenomenon, showing non-Debye behavior. This little broadening of the peak was the outcome of the dispersion of relaxation time with fluctuating time being constant. This constructed plot of *M*″_max_ gave *f_max_*, which is a relaxation frequency, and gave information about the relaxation time (*τ*″ max) of dipoles. *M*″ gave a relaxation peak centering on the dispersive part of *M*′. The frequency range lower than this peak maximum specifies long distance movement of charge carriers, and a higher frequency range overhead this fmax identifies the short distance movement of charge carriers [55]. The frequency dependent *M*″ spectrum was fitted by the Bergman modified Kohlraush, Williams, and Watts (KWW) function, expressed as,

(6) $$M^{\prime \prime }=\frac{M_{max}^{\prime \prime }}{\frac{\left(1-c\right)\;}{\left(a+b\right)}\;\left[b{\left(\frac{f}{f_{max}}\right)}^{-a}+a{\left(\frac{f}{f_{max}}\right)}^{b}\right]+c}$$

(7) $$M^{\prime \prime }=\frac{M_{max}^{\prime \prime }}{\frac{1\;}{2}\;\left[\left(\frac{f_{max}}{f}\right)+\left(\frac{f}{f_{max}}\right)\right]}\;$$

where, *M*″_*max*_ is the peak maximum of *M*″ vs. the frequency plot and *f*_*max*_ is the corresponding frequency, a and b represent two independent peak shape parameters for both low and high frequency regions, respectively, and c signifies the smoothing parameter. The values of these peak shape parameters determine the transition from ideal Debye to non-Debye type. If *a* and *b* are equal to 1 and c is equal to 0, then Equation (5) is reduced to Equation (6), which represents the ideal Debye response and symmetric nature of the *M*″ plot. The values of a and b estimated from fitting are 0.9 and 0.4, respectively. This confirms the presence of a non-Debye type of relaxation process [60]. Figure 9c displays the complex modulus plot formed between *M*″(f) and *M*′(f), which has furnished an evidence concerning the main structure, where the enormous volumetric part was engaged by grain boundaries. The appearance of two overlapping semicircular arcs at low and high frequency regions suggests the presence of two different relaxation phenomena having fewer differences in their relaxation time constants. This also supports our impedance results (as discussed later). The semicircle seen at an elevated frequency shows the capacitive influence of grains on the conductivity of a material.

### 3.8. Impedance Spectroscopy

**Figure 10:**
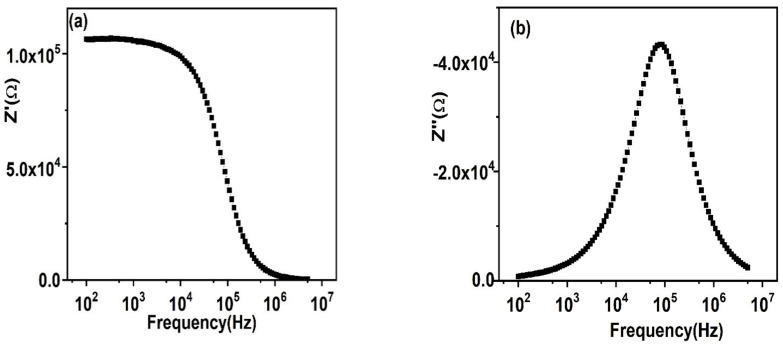
Frequency relying on the performance of (**a**) *Z*′; (**b**) *Z*″ of impedance formalism.

Figure 10a,b show the measured impedance analysis (real and imaginary parts) studied at room temperature along with altering the frequency. It is observed that *Z*′ shows a high value in lesser frequency and, then, a decline appears along with a rising frequency, superposing at the maximum frequency value; thus, giving no more reliance on frequency. The increasing *Z*′ denotes the greater influence of interfacial polarization coming from the dielectric system as mentioned earlier. *Z*′ shows a decline at elevated frequency values and is the outcome of release in the space charge, which also confirms the increased ac conductivity at elevated frequencies. As *Z*″, which is the imaginary part of impedance, is equal to CR^2^, it gives resistance along with a frequency change. The plot shows a clear peak, and, therefore, the diverse relaxation processes are present. This asymmetric and broader peaks having a full width at half maxima (FWHM), more than 1.14 decades proposes that more than one relaxation process is present [61].

**Figure 11:**
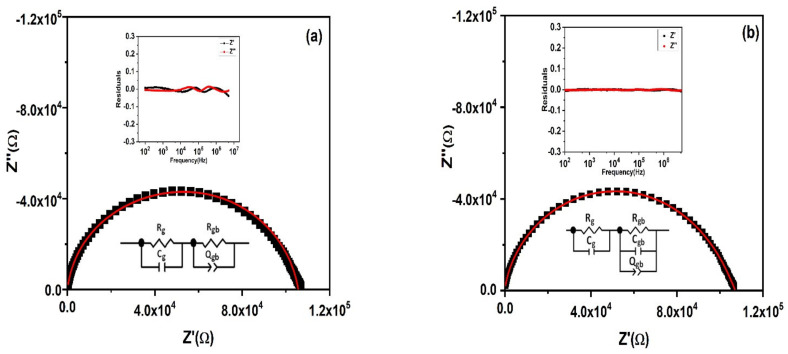
(**a**,**b**) Fitting of complex impedance *Z*″ vs. *Z*′ plot by >(R_g_C_g_) (R_gb_Q_gb_) and (R_g_C_g_) (R_gb_Q_gb_C_gb_) equivalent circuit models along with insets showing the residual plots.

The Nyquist plot is drawn between *Z′* verses *Z*″ is employed to find an influence of grain, grain boundary, as well as electrode contact to the electrical properties of the PbSe sample. The deformed Nyquist plot partaking in the asymmetric semicircular arc with its center sited lower than the real *Z*′ axis is shown in Figure 11. This points toward withdrawal from the ideal Debye type and an indication of multiple relaxation processes. To extract the capacitances and resistances of different electroactive regions of a material, various equivalent circuit models were employed to interpret the complex impedance plane plots, as shown in Figure 11. The impedance data were first fitted by applying the conventional equivalent circuit model (R_g_C_g_) (R_gb_Q_gb_). The constant phase element Q was introduced to address the non-Debye type behavior. The values of different fitted parameters estimated by this model were not physically acceptable with a chi-squared value of 0.001. The frequency dependent residual plot between the measured and fitted data is an alternate approach to check the quality of fit. Residual is the difference between the experimental and fitted curves. The residual plot (inset of Figure 11a) indicates a deviation in the higher frequency region. However, good agreement between the experimental and fitted data was observed by employing an equivalent circuit model comprised of the series combination of (R_g_C_g_) and (R_gb_Q_gb_C_gb_), as shown in Figure 11b. An additional capacitor element was inserted in parallel with the (R_gb_ Q_gb_) equivalent circuit. All fitted parameters extracted from this model were physically acceptable with a minimum chi-square value of 4.46 × 10^−5^. The estimated values of R_g_, R_gb_, C_g_, C_gb_, Q_gb_, and n_gb_ are listed in Table 2.

**Figure 12:**
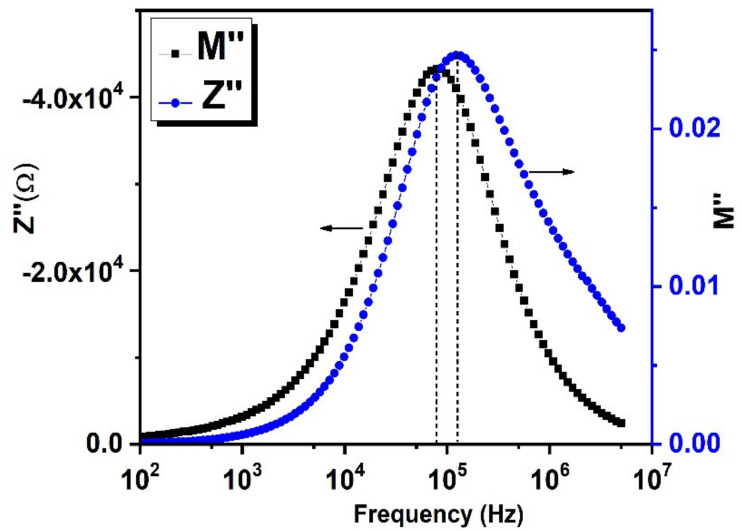
Combined *Z*″ and *M*″ vs. Frequency plot.

The combined frequency dependent *Z*″ and *M*″ spectra could help one to understand the presence of short- or long-range conduction mechanisms in the sample. The overlapping between the peak frequencies of *Z*″ and *M*″ indicates the presence of long-range movement of charge carriers. However, there is a separation between the f_max_ of *Z*″ and *M*″ plot as shown in Figure 12, suggesting the short-range mobility of charge carriers and a departure from the ideal Debye behavior [61].

## 4. Conclusions

Lead (II) diphenyldiselenophosphinate [Pb(iPh_2_PSe_2_)_2_] compound was successfully prepared and confirmed via FTIR, organic elemental analysis, and mass spectrometry. The TGA results for [Pb(iPh_2_PSe_2_)_2_] displayed that the precursor started breakdown around 220 °C and completely decomposed at about 380 °C. The depositions of PbSe obtained by [Pb(iPh_2_PSe_2_)_2_], noticed at 400 and 450 °C, were obtained by utilizing the aerosol-assisted chemical vapor deposition approach. The XRD analysis, SEM images, and TEM micrographs verified the formation of three-dimensionally grown interlocked or aggregated nanocubes of PbSe. The dielectric constant and tan δ showed a decrease, as described by the Koop model and the Maxwell–Wagner approach. The ac conductivity (σac) showed a distinct leaning with an increasing frequency higher than 10 kHz. The jump relaxation model (JRM) provided a good explanation about this behavior. The Jonscher power law was used for fitting of frequency reliant ac conductivity, which gave a frequency exponent s value lower than 1, proposing short-ranged translational motion of charge carriers. The impact of grains plus grain boundaries headed for capacitance and resistance of polycrystalline medium was clarified by Cole–Cole plots and impedance analysis. The electrical properties of PbSe were correlated by proposing a corresponding circuit (R_g_ C_g_) (R_gb_ Q_gb_ C_gb_) to fit the impedance plot. The fitted values of R_g_ and R_gb_ showed that grain boundaries have more resistive nature than grains.

## Figures and Tables

**Table 1:** The ac conductivity fitting parameters by Jonscher’s power law.

σ_dc_ (S/m)	*A*	*s*
9.0148 × 10^−5^	1.4195 × 10^−8^	0.657

**Table 2:** Parameters extracted from ZView fitting of complex impedance plot.

R_g_ (Ω)	C_g_ (F)	R_gb_ (Ω)	C_gb_ (F)	Q_gb_	n_gb_
26,642	1.295 × 10^−11^	80,562	7.422 × 10^−11^	1.361 × 10^−9^	0.654

## Data Availability

The data presented in this study are available on request from the corresponding author.

## Supplementary Materials

The following are available online at https://www.mdpi.com/article/10.3390/nano11112817/s1, Figure S1: Elemental Analysis of Bis(isobutyldithiophosphinato)lead complex, Figure S2: FTIR Analysis of Bis(isobutyldithiophosphinato)lead complex, Figure S3: Mass spectrometry Analysis of Bis(isobutyldithiophosphinato)lead complex, Figure S4: EDX analysis of PbSe at (a) 400 °C, (b) 450 °C.
