# Supplementary material for: Impedance Spectroscopy Analysis of PbSe Nanostructures Deposited by Aerosol Assisted Chemical Vapor Deposition Approach
Source: Nanomaterials (Basel). 2021 Oct 23;11(11):2817. doi: 10.3390/nano11112817 (PMC8622599; doi:10.3390/nano11112817)
Supplement: Supplementary file 1 [file nanomaterials-11-02817-s001.zip › nanomaterials-1296604-supplementary.pdf]

Supplementary Information

Impedance Spectroscopic Analysis of PbSe Nanostructures Deposited by Aerosol Assisted Chemical Vapor Deposition approach

Sadia Iram [1,4], Azhar Mahmood [1*], Muhammad Fahad Ehsan [1], Asad Mumtaz [1], Manzar Sohail[1], Effat Sitara [1],Shehla Mushtaq[1], Syeda Arooj Fatima [2], Rubina Shaheen [2], Nasir Mahmood Ahmad [3], Sajid Nawaz Malik[3], and Mohammad Azad Malik [4]

[1]School of Natural Sciences, National University of Sciences and Technology, Islamabad 44000,Pakistan;sadia.iram@sns.nust.edu.pk(S.I.);dr.azhar@sns.nust.edu.pk(A.M.);m.fahad.ehsan@sns.nust.edu.pk(M.F.E.);asad.mumtaz@sns.nust.edu.pk(A.M.);manzar.sohail@sns.nust.edu.pk (M.S.); effat.sitara@sns.nust.edu.pk (E.S.);shehla.mushtaq@sns.nust.edu.pk(S.M)

[2] Central Diagnostic Laboratory, Physics Division, PINSTECH, P.O. Nilore.Islamabad 45500, Pakistan; syedaarooj80@yahoo.com (S.A.F.); rubina_shahin_2003@yahoo.com (R.S.)

[3] Department of Materials Engineering, School of Chemical and Materials Engineering (SCME)-National University of Sciences and Technology (NUST), Islamabad 44000, Pakistan; Nasir.ahmad@scme.nust.edu.pk (N.M.A); sajidnawaz@scme.nust.edu.pk (S.N.M.)

[4] Department of Materials, University of Manchester, M13 9PL Manchester, UK; azad.malik@manchester.ac.uk (M.A.M.)

*Correspondence: dr.azhar@sns.nust.edu.pk, Tel.: +92-51-9085-5574

Received: date; Accepted: date; Published: date

Mr M JENNINGS
MICRO ANALYTICAL LABORATORY
SCHOOL OF CHEMISTRY
THE UNIVERSITY OF MANCHESTER
MANCHESTER M13 9PL

PLEASE GIVE APPROX. PERCENTAGES OF ELEMENTS PRESENT IN THE EXPECTED COLUMN.

	EXPECTED	FOUND	CHARGE
C	32.27	32.48	
H	2.26	2.22	—
N			
Cl			
Br			
I			
F			
S			
P	6.93	6.93	—
Mol.Wt.	893.41		
Metal	Se = 35.35		
Other	Pb = 23.19	22.90	
		TOTAL CHARGE =	—

COMMENTS

TGA Wt= 4.79mg

Figure S1; Elemental Analysis of $C_{16}H_{36}PbP_2S_4$, Bis(isobutyldithiophosphinato)lead complex

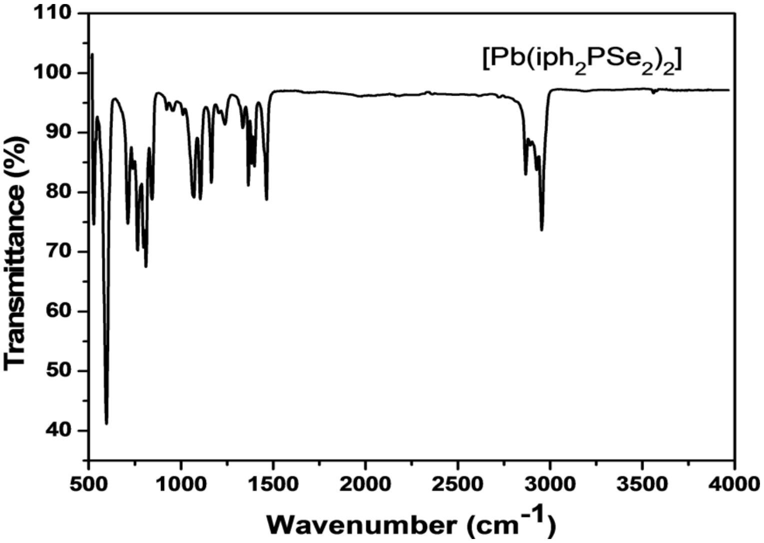

Figure S2; FTIR analysis of $C_{16}H_{36}PbP_2S_4$, Bis(isobutyldithiophosphinato)lead complex

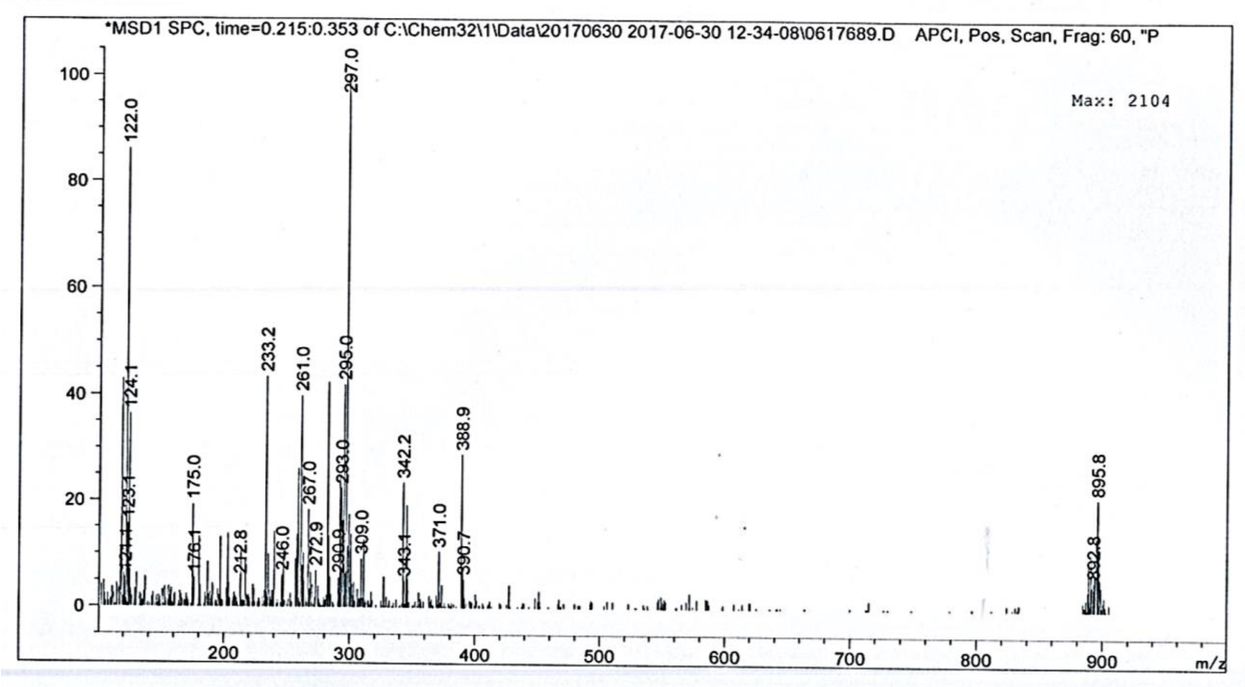

Figure S3; Mass spectrometry analysis of $C_{16}H_{36}PbP_2S_4$, Bis(isobutyldithiophosphinato)lead complex

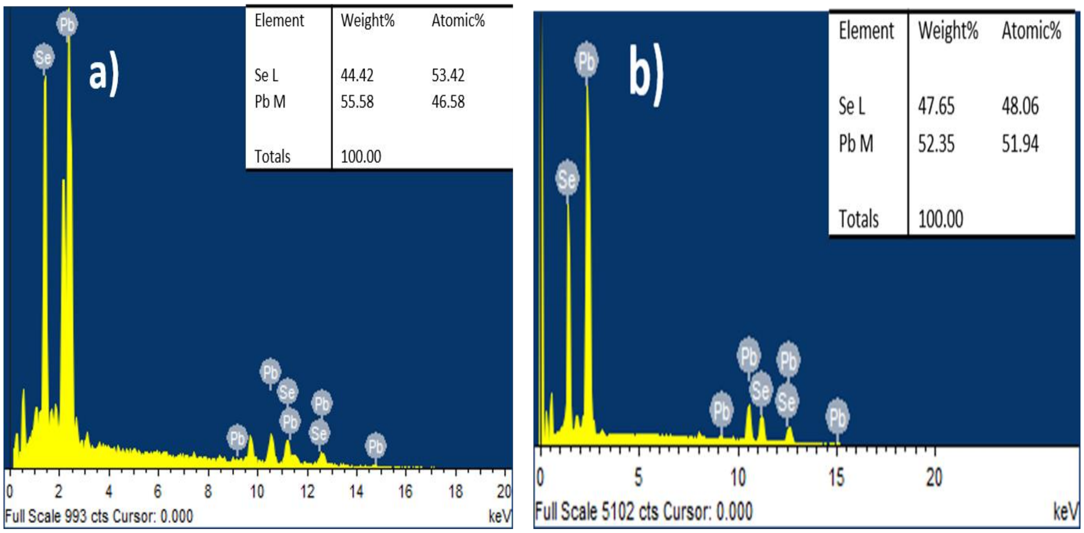

Figure S4; EDX analysis of PbSe at (a) 400 °C, (b) 450 °C
